# CNPS.cycle: streamlining shotgun metagenomic data analysis for biogeochemical cycles

**DOI:** 10.1128/msystems.01021-25

**Published:** 2025-10-22

**Authors:** Zhengfu Yue, Jing Zhang, Wei Xu, Liang Peng, Tianshun Liu, Shoushan Sheng, Ye Tao, Liang Zeng, Zelong Zhao, Daniele Alberoni, Loredana Baffoni, Qiaoyan Zhang, Beibei Liu, Qinfen Li, Jing Zhang, Yukun Zou

**Affiliations:** 1 Key Laboratory of Low-carbon Green Agriculture in Tropical region of China, Ministry of Agriculture and Rural Affairs, Hainan Key Laboratory of Tropical Eco-Circular Agriculture, Environmental and Plant Protection Institute, Chinese Academy of Tropical Agricultural Sciences 117453, Haikou, China; 2 Hainan Danzhou Tropical Agro-ecosystem National Observation and Research Station, Chinese Academy of Tropical Agricultural Sciences 118231 , Danzhou, China; 3 Department of Environmental Sciences, School of Tropical and Laboratory Medicine, Hainan Medical University 12455, Haikou, China; 4 Yancheng Dafeng District Plant Protection Station, Yancheng, China; 5 Shanghai BIOZERON Co., Ltd, Shanghai, China; 6 Department of Agricultural and Food Sciences, University of Bologna (Alma Mater Studiorum) 9296, Bologna, Italy; 7 School of Breeding and Multiplication (Sanya Institute of Breeding and Multiplication), Key Laboratory of Green Prevention and Control of Tropical Diseases and Pests, Ministry of Education (School of Tropical Agriculture and Forestry), School of Environmental Science and Engineering, Hainan University 74629, Haikou, China; Syracuse University, Syracuse, New York, USA

**Keywords:** carbon, nitrogen, phosphorus, sulfur, R package, automated

## Abstract

Shotgun metagenomic data analysis for investigating biogeochemical cycles in the environment remains challenging, primarily due to the steep learning curve, intensive time investment, and high computational demands, all of which pose significant barriers for many researchers. We present a new R package called “CNPS.cycle,” designed to streamline the interpretation of shotgun metagenomic data related to biogeochemical processes, complete with visually informative outputs. This comprehensive package comprises four distinct analysis modules, focused on carbon, nitrogen, phosphorus, and sulfur cycling. Users can easily utilize the package by uploading annotation result files derived from shotgun metagenomic data, specifically those based on the Kyoto Encyclopedia of Genes and Genomes and the NCBI non-redundant protein sequence database. The package then automates essential steps, including data preprocessing, curation, and differential analysis of biogeochemical cycle-related genes; analysis of microorganisms possessing biogeochemical cycle-related genes at the contig level; β-diversity analysis; and, finally, data visualization. The outcome is a comprehensive analysis revealing differentially abundant genes and functionally significant microbial entities associated with the carbon, nitrogen, phosphorus, and sulfur cycles, presented in the form of tables and high-quality images. This tool will provide profound insights into the relationship between soil microorganisms and elemental chemical cycles, thereby advancing our comprehension of soil ecosystems. For accessibility, the CNPS.cycle package is available on GitHub (https://github.com/yuezhengfu/CNPS.cycle), where detailed instructions on its usage can be found in the project’s GitHub page (https://github.com/yuezhengfu/CNPS.cycle/wiki).

**IMPORTANCE:**

The “CNPS.cycle” R package offers significant environmental implications by simplifying the analysis of shotgun metagenomic data related to biogeochemical cycles. Its automated workflow identifies key genes and microbes involved in carbon, nitrogen, phosphorus, and sulfur cycling, enhancing our understanding of microbial contributions to ecosystem functions. This tool enables researchers to explore microbial-mediated nutrient cycling more efficiently, supporting informed decisions in environmental management and climate change mitigation. By providing accessible, high-quality outputs, “CNPS.cycle” facilitates data-driven insights into the interplay between microbes and global biogeochemical processes.

## INTRODUCTION

Biogeochemical cycles are fundamental processes that regulate ecological stability, influence climate patterns, sustain environmental dynamics, and ultimately support life on Earth (1). Among these, the cycling of carbon, nitrogen, phosphorus, and sulfur (CNPS) plays a central role in shaping ecosystem functions across diverse habitats—including soils, sediments, freshwater systems, and marine environments (2–5). Microbial communities are the primary drivers of these cycles, mediating biochemical transformations through the expression of functional genes that encode key enzymes involved in elemental turnover (6–8). Understanding CNPS cycling at the microbial level requires accurate identification of functional genes and their associated taxa from metagenomic data. However, due to the complexity of microbial ecosystems and the vast number of annotated genes in metagenomes, efficiently linking gene functions to biogeochemical processes remains a substantial analytical challenge (9).

Previous research methods for studying elemental cycling genes and associated microorganisms in the environment were complex and demanded high technical proficiency, posing significant challenges for researchers, especially novices. Although Zheng et al. developed a gene microarray method capable of quantitatively detecting 72 genes covering the carbon, nitrogen, phosphorus, and sulfur cycles simultaneously (3), the method lacked the ability to associate detected functional genes with specific microbial taxa, thereby limiting its capacity to identify the microbial contributors driving different biogeochemical pathways. Databases like CCycDB (https://github.com/ccycdb/ccycdb.github.io), NCycDB, PCycDB, SCycDB, and NR (NCBI non-redundant protein sequence database), developed based on shotgun metagenomic technology, effectively linked carbon, nitrogen, phosphorus, and sulfur cycling genes with the microorganisms possessing biogeochemical cycle-related genes (10–12). However, users still need to manually construct complex elemental cycling pathway diagrams and identify the microorganisms possessing biogeochemical cycle-related genes from thousands of NR species annotations. Moreover, most software for analyzing shotgun metagenomic sequences is tailored for Linux systems (13–15), demanding high computational performance and facing issues of high costs and complex software installation dependencies, which deters many users.

Although several tools such as METABOLIC, DRAM, and DiTing have been developed to analyze functional genes from metagenomic data (16–18), none are specifically designed to streamline the interpretation of biogeochemical cycling processes—particularly those related to CNPS. In these tools, users must often manually filter thousands of annotated genes, curate relevant subsets, and infer their association with microbial taxa and elemental cycles. This process requires substantial expertise in both bioinformatics and microbial ecology. By contrast, CNPS.cycle is built to bridge this gap: rather than aiming for exhaustive pathway reconstruction, it focuses on a carefully curated set of key functional genes that represent the most ecologically relevant CNPS processes. It simplifies data processing and highlights the most biologically meaningful trends, enabling researchers to efficiently extract insights from complex metagenomic data sets—even without deep computational backgrounds (Table S1).

CNPS.cycle summarizes 42 elemental cycling processes (including 7 for carbon, 18 for nitrogen, 2 for phosphorus, and 15 for sulfur), selected based on their ecological importance and prevalence in environmental metagenomes (19–24). For each process, representative genes are included to reflect the dominant metabolic transformations. This targeted approach is accompanied by rich visual outputs—including heatmaps and fold change plots—that facilitate intuitive comparisons across experimental groups. In addition, CNPS.cycle links functional genes to their taxonomic origins using user-provided NR annotations, enabling users to explore microbial contributors to each CNPS process at multiple taxonomic levels. The package is platform-independent, easy to install on standard desktop systems, and does not rely on Linux or high-performance computing. With flexible source code and fully documented workflows, CNPS.cycle is designed to support reproducible, customizable analysis for microbial ecologists, biogeochemists, and environmental genomics researchers.

## MATERIALS AND METHODS

### Workflows

CNPS.cycle is an R package developed for the comprehensive analysis of elemental cycling processes. In total, 119 Kyoto Encyclopedia of Genes and Genomes (KEGG) orthology (KO) entries were carefully curated and grouped into 42 representative biogeochemical cycling processes, including 22 KOs for 7 carbon cycling pathways, 48 for 18 nitrogen pathways, 5 for 2 phosphorus pathways, and 47 for 15 sulfur pathways. The complete list of KO entries, along with their corresponding gene names and assigned cycle categories, is provided in Table S2.

**Fig 1:**
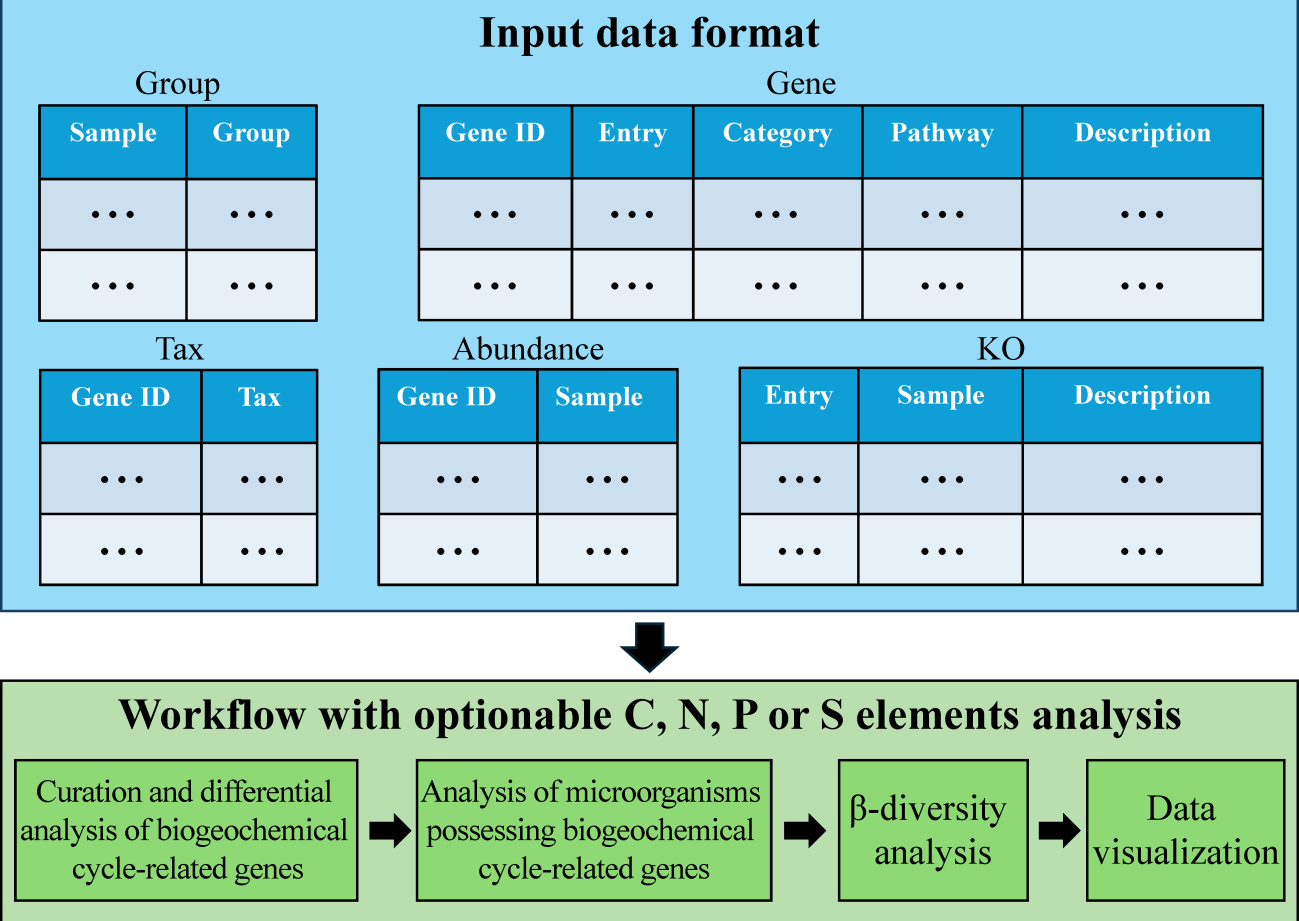
The workflow describing the steps of different elemental analysis modules in CNPS.cycle. In the five input data sets, “Group” represents the sample grouping file, “Gene” represents the mapping file between non-redundant gene sets in shotgun metagenomics and KO entries as well as KEGG PATHWAY, “Tax” represents the BLAST result comparing non-redundant gene sets with the NR (non-redundant protein sequence database), “Abundance” represents the abundance file of non-redundant gene sets in shotgun metagenomics, and “KO” represents the abundance file of KO entries mapped from non-redundant gene sets in shotgun metagenomics.

CNPS.cycle encapsulates a total of 57 functions, organized into four separate analytical modules: carbon, nitrogen, phosphorus, and sulfur cycles. Within each module, CNPS.cycle provides four distinct functional analyses, including curation and differential analysis of biogeochemical cycle-related genes, analysis of microorganisms possessing biogeochemical cycle-related genes, β-diversity analysis, and data visualization (Fig. 1).

### Input data requirements

Since the input files required by CNPS.cycle are derived from downstream metagenomic analyses, users are recommended to first complete standard preprocessing steps. Raw sequencing reads should be assembled using metagenomic assemblers such as metaSPAdes, MEGAHIT, or IDBA-UD (25–27). Gene prediction can then be performed on the assembled contigs using MetaGeneMark, Prodigal, or Glimmer-MG (28–30).

For functional and taxonomic annotation, predicted amino acid sequences should be aligned using KofamScan with its KEGG-based HMM profile database for functional annotation, and DIAMOND against the NCBI NR database for taxonomic classification (15, 31). The resulting annotation files, along with associated gene abundance profiles, serve as inputs for CNPS.cycle.

Specifically, CNPS.cycle requires the following five tab-delimited input tables:

- KO table (ko): a gene abundance matrix based on KO annotation. Rows represent KO identifiers (e.g., K00174), and columns represent individual samples. The first column contains KO IDs, and the last column is a “Description” column providing the functional annotation of each KO. All intermediate columns contain numeric abundance values.
- Group table (group): a simple table mapping each sample to its corresponding experimental group. It contains two columns: SampleID and Group, where Group indicates the biological or treatment group.
- Gene annotation table (Gene): this table maps non-redundant gene IDs to their KO annotations. It must contain at least two columns: GeneID and KO (also referred to as Entry). This file is used to associate gene-level abundance with functional-level (KO-level) information.
- Taxonomy annotation table (tax): this table provides taxonomic information for each gene. It must contain at least two columns: GeneID and Taxonomy, which map each gene to its corresponding taxonomic classification based on NR annotation.
- Gene abundance table (abundance): a matrix of abundance values for each gene (non-redundant gene set) across samples. The first column is GeneID, and subsequent columns are sample IDs with numeric values indicating gene abundance.

Example files for each input type are included in the built-in test data set and are available in the GitHub repository (https://github.com/yuezhengfu/CNPS.cycle/tree/main/data). Users can download the example data set with .RData suffix, and open it directly in RStudio to inspect the structure and content of the required input files. All files must use consistent sample IDs across tables.

### Curation and differential analysis of biogeochemical cycle-related genes

KO-level abundance values are extracted from the user-provided KO abundance matrix and summarized into pathway-level abundance profiles using dedicated functions for each element: Ccyc.abundance(), Ncyc.abundance(), Pcyc.abundance(), and Scyc.abundance(). These functions calculate the cumulative abundance of genes associated with each defined biogeochemical sub-process.

To ensure robust and statistically sound differential analyses, CNPS.cycle implements the following statistical procedures: (i) Shapiro-Wilk test to assess data normality; (ii) for two-group comparisons, both parametric (*t*-test) and non-parametric (Wilcoxon rank-sum test) methods are applied; (iii) for comparisons across more than two groups, one-way analysis of variance and Kruskal-Wallis tests are used. In addition, CNPS.cycle supports multivariate community-level analyses using Bray-Curtis dissimilarity matrices with permutational multivariate analysis of variance (PERMANOVA) (Adonis), analysis of similarities, and multiple response permutation procedure. Additionally, the fold.change() function is applied for pairwise differential analysis, generating fold change value tables to identify significantly enriched or depleted pathways across groups. All statistical results are exported as structured tab-delimited text files to facilitate downstream visualization and interpretation.

To achieve adequate statistical power and minimize false negatives, we recommend a minimum of *n* ≥ 3 biological replicates per group. This threshold ensures the reliability and sensitivity of statistical tests used for detecting differential pathways, regardless of whether parametric or non-parametric methods are applied.

### Analysis of microorganisms possessing biogeochemical cycle-related genes

The presence of each elemental cycling process in the input data set is determined based on two criteria: (i) non-zero KO-level abundance across samples, and (ii) successful mapping of at least one KO associated with the process to the gene annotation table. CNPS.cycle implements this process via internal helper functions, including Ccyc.host(), Ncyc.host(), Pcyc.host(), and Scyc.host(), which return binary vectors indicating the presence or absence of detectable processes. For data sets in which a given process is detected, CNPS.cycle assigns taxonomic information to the genes carrying the corresponding KOs by integrating two user-provided annotation sources. The first is a functional annotation table (Gene) that links each gene to its KO assignment, thereby enabling the identification of genes involved in carbon, nitrogen, phosphorus, and sulfur cycling. The second is a taxonomic annotation table (tax), generated through NR-based alignment, which associates each gene with taxonomic classifications from phylum to species level. By matching gene identifiers across these two tables, CNPS.cycle establishes the relationship between biogeochemical functions and microbial taxa across six taxonomic ranks—phylum, class, order, family, genus, and species. The tool summarizes the taxonomic composition associated with each detected sub-process and outputs the results as .csv files.

### β-Diversity analysis

CNPS.cycle provides a unified framework for assessing β-diversity in microbial communities involved in carbon, nitrogen, phosphorus, and sulfur cycling. For each cycle, KO-level gene abundance matrices across samples are used to calculate Bray-Curtis dissimilarities. These are computed using the pcoa.arg() function, and statistical differences among sample groups are assessed via PERMANOVA. Ordination is performed using principal coordinates analysis (PCoA), principal component analysis (PCA), and non-metric multidimensional scaling (NMDS) via pcoa.arg(), pca.arg(), and nmds.arg() functions, respectively. NMDS analyses also include stress values as indicators of model performance. All outputs—including dissimilarity matrices, ordination coordinates, and statistical test results—are automatically saved to structured directories under each elemental module.

### Data visualization

CNPS.cycle incorporates multiple data visualization functionalities to support interpretation of elemental cycling patterns. For each module, the package generates publication-ready visual outputs, including (i) heatmaps: displaying the relative abundance of biogeochemical cycling genes or associated microorganisms across samples or groups at multiple taxonomic levels (phylum to species), facilitating comparative analysis of functional potential and revealing key microbial contributors to specific cycling sub-processes; (ii) differential abundance plots: highlighting fold changes and statistical significance of key genes or taxa between groups; (iii) ordination plots: including PCA, PCoA, and NMDS, to illustrate community-level differences in biogeochemical functionality; and (iv) pathway diagrams: overlaying gene abundance heatmaps onto curated element-specific cycle diagrams (carbon, nitrogen, phosphorus, sulfur) to visually represent key sub-processes with detected activity, enabling intuitive interpretation of complex biogeochemical pathways. All plots are generated using built-in functions in CNPS.cycle and exported in high-resolution PDF format, organized by element and output type.

### Simulated metagenomic data generation and functional annotation workflow

To systematically evaluate the performance of functional annotation tools for biogeochemical cycling genes, we generated simulated metagenomic data sets with known composition. Specifically, we selected 83 KO identifiers associated with carbon, nitrogen, phosphorus, and sulfur cycling pathways from the KEGG database (https://www.genome.jp/kegg/), representing a broad set of environmental metabolic functions. For each KO, the corresponding representative nucleotide and protein sequences were retrieved from KEGG and used as reference templates.

Using InSilicoSeq (v.2.0.1), five synthetic shotgun metagenomic data sets were generated, each comprising 8 million paired-end reads. The simulation incorporated realistic sequencing noise and abundance variation to reflect typical complexities observed in environmental metagenomic data. The simulated data sets have been deposited in the NCBI Sequence Read Archive under BioProject accession number PRJNA1285331, and detailed information regarding KO composition is provided in Table S3.

Before CNPS.cycle analysis, raw reads were first subjected to quality control using fastp (v.0.24.0), which removed low-quality reads and adapter contamination. High-quality reads were assembled into contigs using MEGAHIT (v.1.2.9) with a minimum contig length of 500 bp. Open reading frames were predicted from assembled contigs using Prodigal (v.2.6.3) in metagenomic mode to obtain protein-coding gene sequences.

The predicted amino acid sequences were annotated against the KEGG HMM profile database using KofamScan and taxonomically classified by DIAMOND (v.2.1.10) against the NR database (15, 31). To estimate gene abundance, high-quality reads were mapped back to the assembled contigs using Bowtie2 (v.2.4.5), and coverage information was extracted with Samtools (v.1.17). Gene abundance was quantified using transcripts per million (TPM) normalization, which accounts for both gene length and sequencing depth, enabling direct comparison of gene abundances across samples. The resulting functional annotation files, together with TPM-normalized gene abundance profiles, were used as inputs for CNPS.cycle.

In parallel, we applied METABOLIC (v.4.0) and DRAM (v.1.5.0) to the same assembled contig data sets using default parameters to extract functional profiles related to C, N, P, and S cycling pathways. Additionally, DiTing (v.0.95) was applied directly to the raw paired-end reads to generate functional annotations without assembly, allowing comparison between assembly-based and assembly-free approaches.

All output files from CNPS.cycle, METABOLIC, DRAM, and DiTing were processed using the “tidyverse” package in R for data cleaning, statistical analysis, and visualization. Specifically, false positive rates (FPRs), false negative rates (FNRs), and annotation accuracy metrics were calculated and visualized to comprehensively compare the performance and reliability of these annotation tools.

### Computational resources and performance benchmarks

The computational performance of CNPS.cycle depends primarily on data set size, especially the number of genes and samples. To provide practical guidance, we benchmarked the complete CNPS.cycle workflow on a workstation equipped with an Intel i5-14600MF CPU (14 cores, 3.5 GHz) and 64 GB of RAM, running the Windows 11 operating system. For a representative data set comprising approximately 1,048,575 non-redundant genes across nine samples, the full CNPS.cycle pipeline—including functional and taxonomic integration, differential analysis, taxonomic profiling, β-diversity assessment, and data visualization—completed in approximately 3 minutes. For smaller data sets (e.g., fewer than 200,000 genes), analysis typically completes within 1 minute on standard desktop computers with at least 8 CPU cores and 16 GB of memory. All tests were performed using R version 4.3.2 under Windows.

### Access and usage

We have made the CNPS.cycle package publicly available via GitHub at https://github.com/yuezhengfu/CNPS.cycle. The GitHub repository provides instructions for package installation (https://github.com/yuezhengfu/CNPS.cycle/blob/main/README.md), comprehensive usage guidelines (https://github.com/yuezhengfu/CNPS.cycle/wiki/Install-and-usage), test data sets (https://github.com/yuezhengfu/CNPS.cycle/releases/download/V1.0.0/Sample.data.zip), sample scripts (https://github.com/yuezhengfu/CNPS.cycle/wiki/Install-and-usage), and sample output format (https://github.com/yuezhengfu/CNPS.cycle/releases/download/V1.0.0/SampleData_AutomatedExecutionScript.Rmd). Any issues or suggestions users encountered while using CNPS.cycle could be reported through https://github.com/yuezhengfu/CNPS.cycle/issues. We were committed to actively improving CNPS.cycle based on user feedback.

## RESULTS

### Verification of CNPS.cycle

To rigorously assess the reliability, accuracy, and computational efficiency of CNPS.cycle, we constructed five sequence files with known gene annotations from NCBI, then performed functional annotation using CNPS.cycle, METABOLIC, DRAM, and DiTing. By comparing the results, we found that CNPS.cycle outperformed the other software tools in multiple aspects.

**Fig 2:**
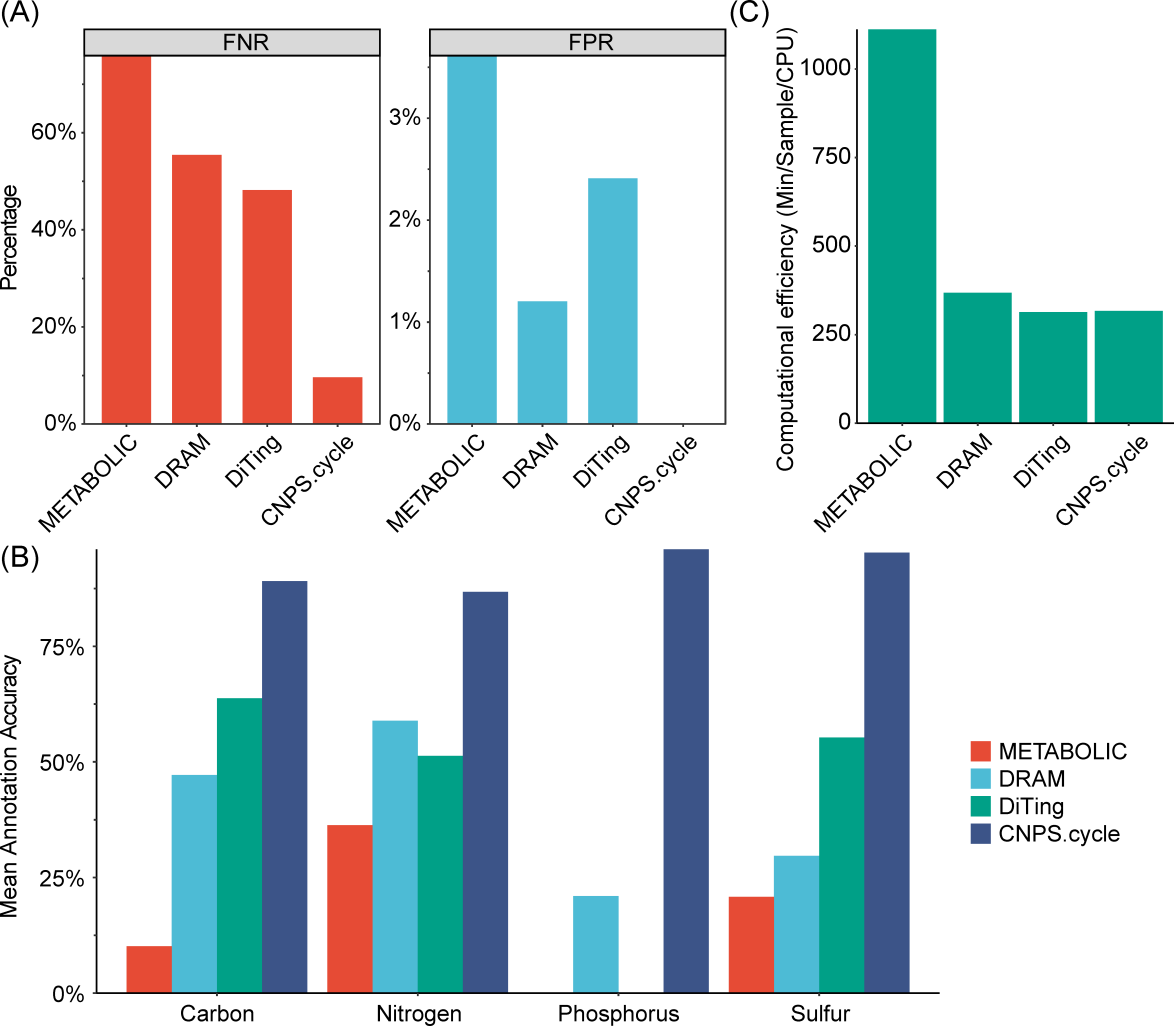
Performance evaluation of CNPS.cycle and three other metagenomic annotation tools. (**A**) Comparison of FNR and FPR. (**B**) Mean annotation accuracy across four biogeochemical cycles: carbon, nitrogen, phosphorus, and sulfur. (**C**) Computational efficiency measured as minutes per sample per CPU.

CNPS.cycle exhibited the lowest FNR and FPR among the evaluated tools (Fig. 2A). CNPS.cycle had an exceptionally low FNR (<10%), whereas METABOLIC, DRAM, and DiTing exhibited significantly higher FNRs (>45%). Regarding FPR, CNPS.cycle also demonstrated optimal performance, whereas METABOLIC exhibited the highest FPR (approximately 3.6%), followed by DiTing (approximately 2.4%) and DRAM (approximately 1.2%).

Moreover, CNPS.cycle exhibited substantial superiority, achieving mean annotation accuracies exceeding 85% across all four assessed biogeochemical cycles (Fig. 2B). Conversely, METABOLIC displayed the lowest performance, with annotation accuracies consistently below 30% across all cycles. The annotation tools DRAM and DiTing demonstrated intermediate yet differential performance patterns. Specifically, DRAM showed slightly higher accuracy than DiTing in annotating nitrogen (approximately 60%) and phosphorus (approximately 20%) cycles, whereas DiTing exhibited a modest advantage over DRAM in annotating carbon (approximately 60%) and sulfur (approximately 55%) cycles. Nevertheless, it is noteworthy that the peak accuracies observed for both DRAM and DiTing (approximately 60%) remained significantly below the minimal accuracy (approximately 85%) achieved by CNPS.cycle.

In addition to its high annotation accuracy, CNPS.cycle also exhibited superior computational efficiency (Fig. 2C). CNPS.cycle processed each sample with a significantly lower computational load compared to METABOLIC, which required more than 1,000 computational units. In contrast, DRAM and DiTing required under 300 computational units. Notably, CNPS.cycle performed similarly to DiTing, making it highly efficient and on par with other leading tools in terms of processing time.

Overall, these results support CNPS.cycle as a robust and accurate tool for biogeochemical cycle analysis. Its capability to provide comprehensive and precise functional annotations, coupled with computational efficiency, highlights its potential value for investigating microbial communities and their ecological functions.

### Application example of CNPS.cycle

Building upon the above validation, we applied CNPS.cycle to a real-world environmental data set derived from a pig manure composting study investigating microbial community dynamics and antibiotic resistance (32). Samples were collected on days 1, 17, and 30 of the composting process to demonstrate the tool’s functionality. Pig manure composting represents a prototypical organic waste treatment system characterized by complex microbial succession and intricate biogeochemical cycling of key elements such as carbon, nitrogen, phosphorus, and sulfur (33). Due to its high nutrient content and dynamic physicochemical conditions, this system provides an ideal model for exploring multi-element cycling mediated by microbial communities (34). Applying CNPS.cycle to this data set enables comprehensive elucidation of the temporal shifts in elemental cycling-related genes and associated microbiota, thereby illustrating the package’s capability to dissect elemental cycling processes in ecologically and agriculturally relevant environments.

**Fig 3:**
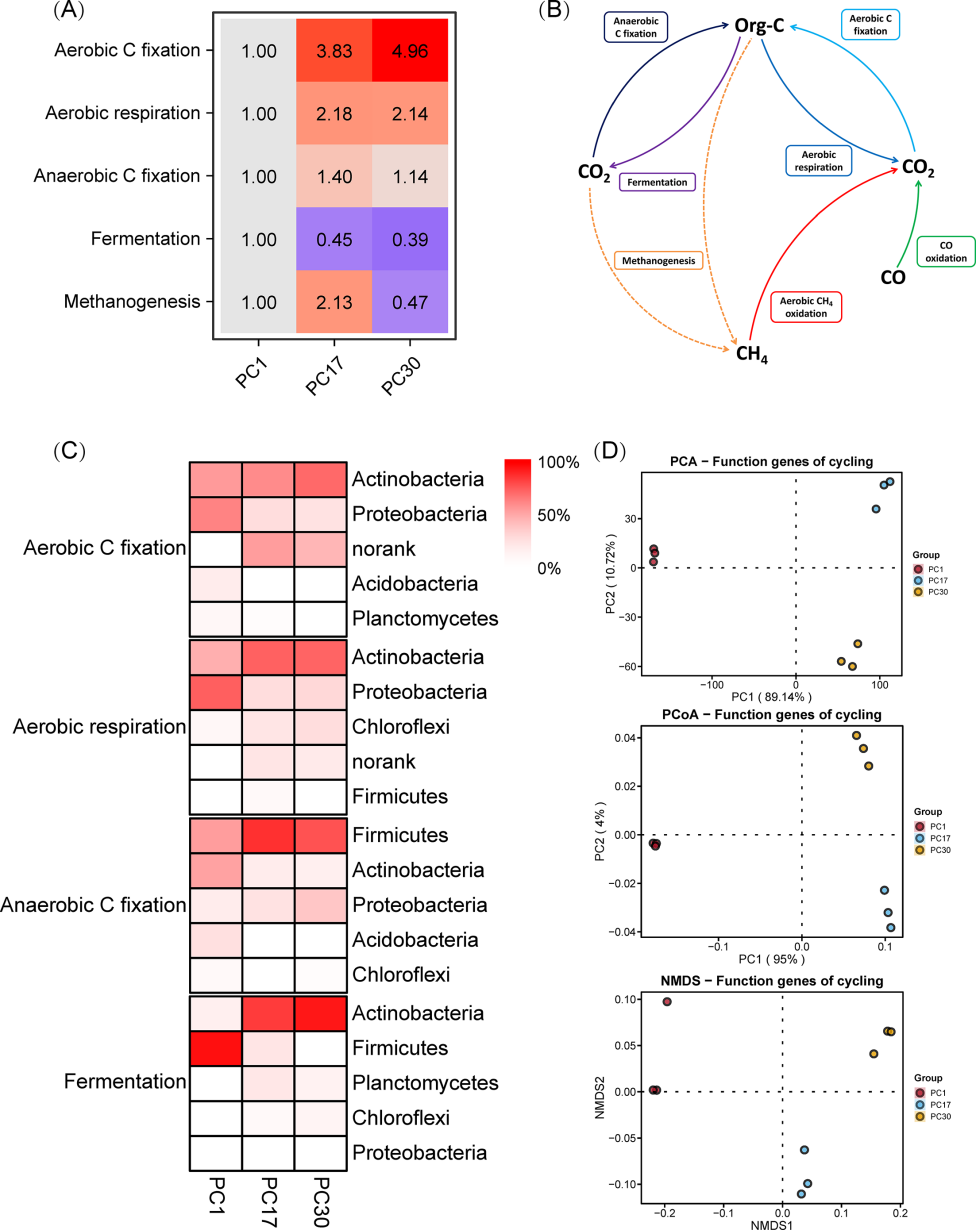
Carbon cycle analysis and visualization results. (**A**) Heatmap depicting fold change in carbon-cycling gene abundance. (**B**) Schematic representation of the carbon cycling pathways. (**C**) Heatmap depicting host microbial composition of carbon cycle functional genes. (**D**) Dimensionality reduction displays of β-diversity of carbon cycle functional genes.

**Fig 4:**
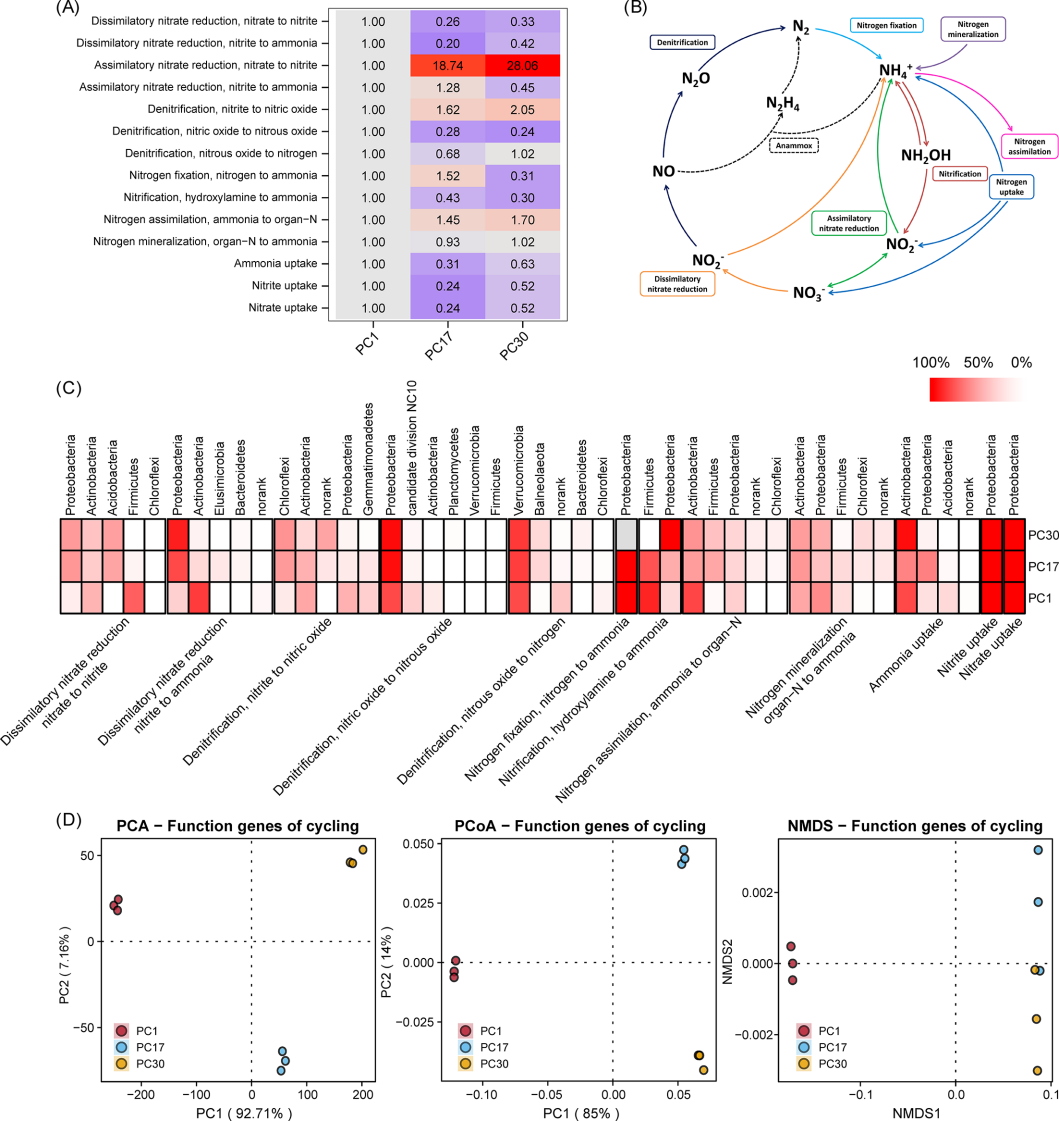
Nitrogen cycle analysis and visualization results. (**A**) Heatmap depicting fold change in nitrogen-cycling gene abundance. (**B**) Schematic representation of the nitrogen cycling pathways. (**C**) Heatmap depicting host microbial composition of nitrogen cycle functional genes. (**D**) Dimensionality reduction displays of β-diversity of nitrogen cycle functional genes.

**Fig 5:**
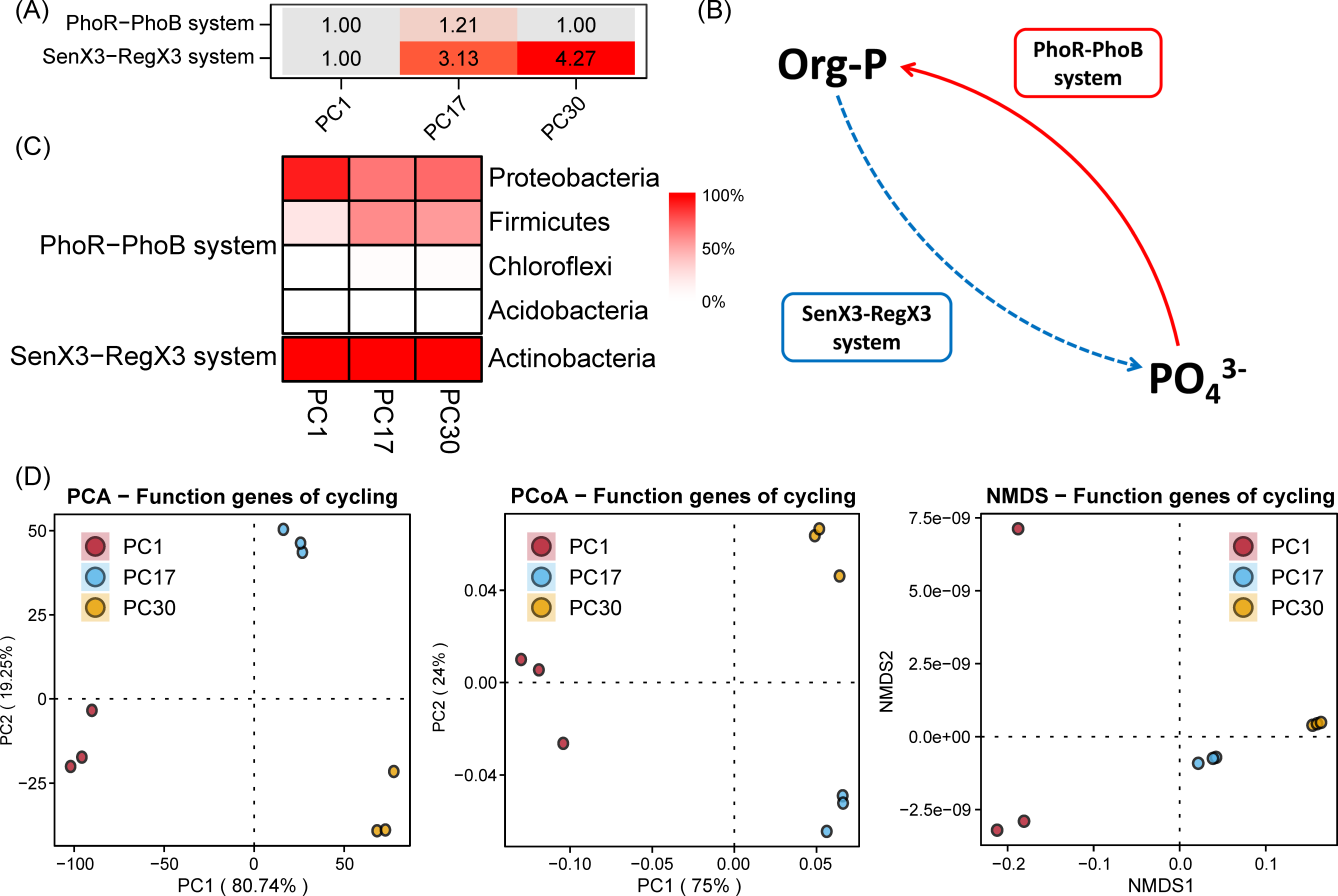
Phosphorus cycle analysis and visualization results. (**A**) Heatmap depicting fold change in phosphorus-cycling gene abundance. (**B**) Schematic representation of the phosphorus cycling pathways. (**C**) Heatmap depicting host microbial composition of phosphorus cycle functional genes. (**D**) Dimensionality reduction displays of β-diversity of phosphorus cycle functional genes.

**Fig 6:**
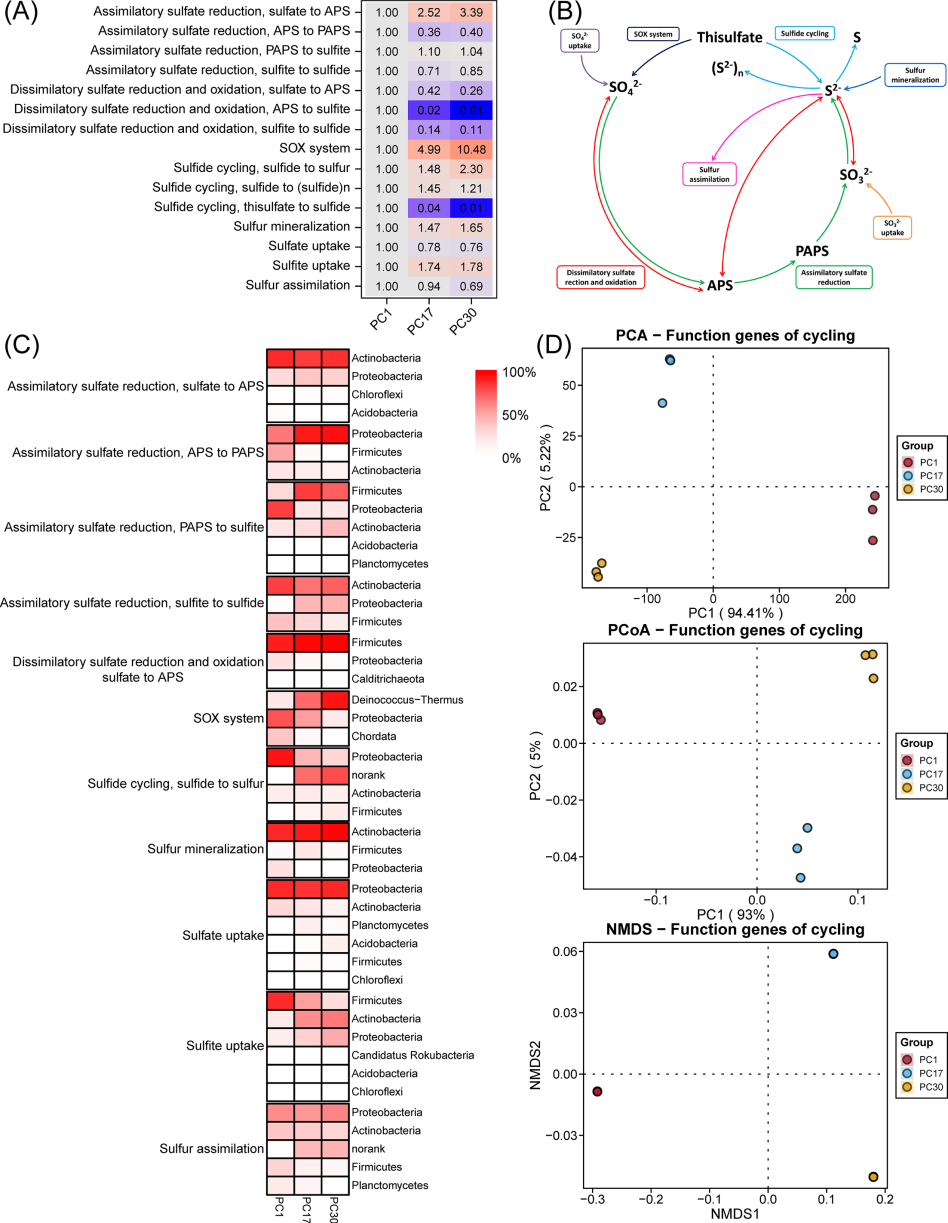
Sulfur cycle analysis and visualization results. (**A**) Heatmap depicting fold change in sulfur-cycling gene abundance. (**B**) Schematic representation of the sulfur cycling pathways. (**C**) Heatmap depicting host microbial composition of sulfur cycle functional genes. (**D**) Dimensionality reduction displays of β-diversity of sulfur cycle functional genes.

The generated folders and files are organized in the final output directory with a specific structure:

- Results: this is the root folder, containing four sub-folders for Carbon, Nitrogen, Phosphorus, and Sulfur.

- Carbon: this folder encompasses the outcomes related to carbon cycling processes and is subdivided into three main categories:

- β-diversity: within this folder, you will find the Bray-Curtis distance matrix files for carbon cycling genes, as well as the results of multivariate analysis of variance based on the Bray-Curtis distance matrix. These are stored within a sub-folder named “Distance.” Additionally, this folder contains visualizations for PCoA, PCA, and NMDS (Fig. 3D), each housed in separate sub-folders named “PCoA,” “PCA,” and “NMDS.” The results consistently indicated that there were significant differences in the carbon cycling functional genes at different time points of compost (Fig. 3D).
- Gene: this folder houses the profile files for carbon cycling genes, located in a sub-folder named “Abundance.” It also includes results for fold change analysis and carbon cycling model diagrams (Fig. 3B), stored in a sub-folder named “Cycle image.” Furthermore, this folder features heat map displays and variance analysis results (Fig. 3A), each situated within sub-folders named “Heatmap.” The results indicated that pig manure composting promoted the abundance of aerobic carbon fixation and aerobic respiration genes while inhibiting the abundance of fermentation genes. In contrast, the abundance of anaerobic carbon fixation and methanogenesis genes initially increased and then decreased over time (Fig. 3A and B). This suggests that our pig manure composting process transitioned from an initial stage of mixed anaerobic and aerobic conditions to a predominantly stable aerobic composting environment primarily driven by aerobic microorganisms. This is a typical dynamic process of organic waste composting. At the beginning of composting, the compost pile usually has a high-moisture content. This high-humidity environment restricts oxygen penetration, leading to anaerobic conditions within the pile. Under these conditions, anaerobic microorganisms, such as methanogens, become active. They decompose organic matter through fermentation and methanogenesis. As composting progresses, aerobic microorganisms begin to thrive in the oxygen-rich environment at the surface of the pile. They decompose organic matter through aerobic respiration, generating a significant amount of heat, which promotes water evaporation and reduces the moisture content of the pile. This makes it easier for oxygen to penetrate deeper into the pile. At this stage, the activity of aerobic microorganisms, such as aerobic bacteria, increases significantly, while the activity of anaerobic microorganisms gradually decreases (35, 36).
- Host_relative_Group: within this folder, there are seven sub-folders, each named after a specific carbon cycling process. Each of these sub-folders contains relative abundance tables and heat map displays at various taxonomic levels (phylum, class, order, family, genus, and species) for the microorganisms participating in the respective carbon cycling process (Fig. 3C). The results indicated that different carbon cycling processes in pig manure composting were dominated by different key microorganisms. Aerobic carbon fixation and aerobic respiration processes were primarily associated with Actinobacteria, while anaerobic carbon fixation was mainly associated with Firmicutes. Composting time promoted the proliferation of these microorganisms. During the early stages of composting, the fermentation process was mainly driven by Firmicutes, whereas in the later stages, it was primarily driven by Actinobacteria (Fig. 3C). Actinobacteria and Firmicutes are the dominant bacterial phyla in composting systems (37). Actinobacteria are a group of highly active microorganisms in aerobic environments. They possess a strong capability to decompose complex organic materials (38). Some members of the Firmicutes phylum exhibit strong survival capabilities under low-oxygen or anaerobic conditions. These microorganisms can thrive in high-moisture content and low-oxygen penetration environments present during the early stages of composting, contributing significantly to the degradation of organic matter (39).

- Nitrogen, Phosphorus, and Sulfur: these folders serve as analogous containers for the results associated with nitrogen, phosphorus, and sulfur cycling processes (Fig. 4 and 5 and Fig. 6), following a structure akin to that of the Carbon folder.

This organization ensures that the outputs of CNPS.cycle are stored for each elemental cycling category, facilitating comprehensive data management and analysis.

It is important to emphasize that while the pig manure composting system serves as a representative application, CNPS.cycle is designed to be broadly applicable to metagenomic data sets from various environmental matrices. The tool’s flexible input structure and modular workflow allow it to be applied to soils, sediments, aquatic systems, extreme environments, or any ecological context where biogeochemical cycles are of interest.

## DISCUSSION

CNPS.cycle represented a significant advancement in shotgun metagenomics technology and biogeochemical cycle research. Its low-barrier technical features and automated analysis workflow were set to leave a lasting impression. The successful installation of CNPS.cycle was a crucial first step. To ensure this, we conducted stability tests on the installation of CNPS.cycle across different R versions in a Windows environment. Our tests spanned R versions 3.5.0 to 4.4.0, revealing that versions R < 4.2.0 encountered numerous dependency installation failures. Therefore, we strongly recommend that users employ the updated version of R and install it via the local installation method we provided. This resolved a large number of compatibility issues related to dependent packages, thereby significantly enhancing the success rate of CNPS.cycle installation. Once CNPS.cycle was successfully installed, users could download the all-in-one automated analysis workflow R markdown (https://github.com/yuezhengfu/CNPS.cycle/releases/download/V1.0.0/SampleData_AutomatedExecutionScript.Rmd). By clicking “Run all” in the RStudio interface, the workflow automatically performed carbon, nitrogen, phosphorus, and sulfur cycle analyses using the built-in data sets.

Accurately and comprehensively reflecting biogeochemical cycling processes was key to the popularity of the CNPS.cycle package. To achieve this goal, we not only integrated the elemental cycling processes provided by DiTing, CcycDB, NCycDB, PCycDB, and SCycDB (11, 40, 41), but also carried out more detailed subdivisions of some important elemental cycling processes. For example, we detailed the nitrogen uptake process by subdividing it into ammonium uptake, nitrite uptake, and nitrate uptake processes. Extensive evidence indicated that soil microorganisms, including bacteria and fungi, widely possessed ammonium transporters, nitrate transporters, and nitrite transporters, enabling them to absorb and transport ammonium, nitrate, and nitrite from the soil into their cells for metabolism and growth (42, 43). Such fine-grained coverage underlies the robustness of CNPS.cycle, leading to consistently low FNR and FPR and high annotation accuracy. The comprehensiveness of CNPS.cycle was also reflected in its statistical analysis capabilities, which covered all data requirements. It supported both parametric and non-parametric tests and automatically selected the appropriate statistical method based on the number of independent groups, ensuring the accuracy and reliability of the analysis results. Additionally, CNPS.cycle not only delved deeply into elemental cycling genes but also identified the microorganisms possessing biogeochemical cycle-related genes. This facilitated researchers’ understanding of the roles and contributions of microorganisms in biogeochemical cycles. CNPS.cycle exhibited superior performance compared to METABOLIC, DRAM, and DiTing in both annotation accuracy and computational efficiency. By providing more precise functional annotations alongside reduced computational demands, CNPS.cycle represents a significant advancement for high-throughput biogeochemical cycle studies.

While CNPS.cycle offers a rapid and effective solution for contig-level functional profiling of microbial contributions to biogeochemical cycles, several limitations remain that warrant future development. First, the tool is specifically designed for shotgun metagenomic data sets, focusing on fragmented contigs rather than complete isolate genomes or high-quality metagenome-assembled genomes (MAGs). As such, CNPS.cycle does not currently support genome-resolved metabolic reconstruction or fine-scale functional annotation. Although beyond the present scope, we recognize that incorporating MAG-level analysis would substantially expand the tool’s applicability to diverse research contexts. Second, the current implementation requires users to complete all upstream processing steps, including sequence quality control, assembly, gene prediction, and abundance estimation, within a Linux-based computing environment. This technical prerequisite imposes considerable barriers for researchers without dedicated bioinformatics expertise or infrastructure. To overcome this limitation, we are developing a web-based, integrated platform that will streamline both upstream processing and downstream functional analysis through an accessible online interface, substantially lowering the technical threshold for broader adoption. Third, CNPS.cycle currently targets four major elemental cycles—carbon, nitrogen, phosphorus, and sulfur. Future versions will extend this scope to include additional ecologically relevant elements, such as iron and arsenic, which are central to microbial-driven redox processes in many environments. We also plan to incorporate viral sequence analysis, enabling taxonomic profiling, lifestyle prediction, and virus-host interaction network construction. Finally, to enhance ecological interpretation, we will integrate environmental metadata into CNPS.cycle and provide advanced analytical modules, including correlation analysis, machine learning-based prediction (e.g., random forests), and structural equation modeling. These features will enable users to explore mechanistic links between environmental factors and microbial elemental cycling with greater precision. Together, these improvements aim to expand CNPS.cycle’s analytical capabilities, reduce technical barriers, and establish a comprehensive, user-friendly platform for investigating the microbial basis of biogeochemical processes across diverse ecosystems.

## Data Availability

The built-in data originates from 9 metagenomic sequence data of pig manure compost published in NCBI (SRR15110374–SRR15110377, SRR15110379–SRR15110380, SRR15110384–SRR15110386), with the BioProject accession number PRJNA746005. These samples were collected at different composting times from 3 pig manure heaps. In addition, we constructed simulated data sets, which have been deposited in the NCBI Sequence Read Archive under BioProject accession number PRJNA1285331. CNPS.cycle is publicly available to all researchers on GitHub (https://github.com/yuezhengfu/CNPS.cycle/), and users can download and install it in R (version ≥4.2.0).

## SUPPLEMENTAL MATERIAL

Supplemental tables msystems.01021-25-s0001.xlsx Tables S1 to S3.

ASM does not own the copyrights to Supplemental Material that may be linked to, or accessed through, an article. The authors have granted ASM a non-exclusive, world-wide license to publish the Supplemental Material files. Please contact the corresponding author directly for reuse.
